# Leaf transcriptome differences between diploid and tetraploid bahiagrass

**DOI:** 10.1002/tpg2.70212

**Published:** 2026-02-28

**Authors:** Maricel Podio, Danilo Fabrizio Santoro, Carolina Marta Colono, Juan Pablo A. Ortiz, Emidio Albertini, Silvina Claudia Pessino

**Affiliations:** ^1^ Instituto de Investigaciones en Ciencias Agrarias de Rosario (IICAR‐CONICET‐UNR) Rosario Argentina; ^2^ Dipartimento di Scienze Agrarie, Alimentari e Ambientali Università degli Studi di Perugia Perugia Italy

## Abstract

Polyploid individuals of the subtropical forage grass *Paspalum notatum* Flüggé (bahiagrass) exhibit distinct phenotypes, including apomixis, enhanced vigor, gigas effects, and increased stress tolerance. While apomixis‐based breeding programs supported by molecular tools have improved agronomic traits such as growth habit, forage dry matter, and lipid profile, a genome‐wide understanding of ploidy‐induced transcriptomic changes is still lacking. In this study, we aimed to generate a comprehensive reference catalog of transcripts differentially expressed in the leaves of diploid and tetraploid individuals, characterize genome responses to polyploidy, and identify candidate genes for breeding. Our results reveal distinct transcriptomic profiles in polyploids, with significant impacts on development, redox homeostasis, and photosynthesis—patterns consistent with those observed in other species. Gene ontology enrichment analyses highlighted key categories related to stress responses and signaling pathways. We also identified critical breeding targets, including transcription factors and hormone‐related genes. Co‐expression network analysis uncovered 532 master regulators affected by genome doubling, with non‐random distribution across the genome and hotspot clustering in specific chromosomes. Overall, our findings provide novel insights into the molecular consequences of polyploidy in *P. notatum*, offering a valuable resource for molecular breeding programs aimed at improving stress tolerance, vigor, and other desirable traits.

## Core Ideas

- Polyploid bahiagrass plants show enhanced adaptation, including stress tolerance and increased vigor.
- Leaf transcriptomes were assembled for sexual diploid and tetraploid bahiagrass plants.
- Transcriptomes reveal how sexual diploid and tetraploid bahiagrass differ at the gene expression level.
- Differentially expressed genes include key breeding targets, such as transcription factors and hormone‐related genes.
- This study provides molecular resources to improve bahiagrass stress tolerance, vigor, and other agronomic traits.

## Plain Language Summary

Bahiagrass plants have more than two sets of chromosomes. These plants, known as polyploids, often grow more vigorously and cope with stress more effectively due to changes in gene expression. In this study, we compared the leaf gene expression of plants with two and four sets of chromosomes to understand how genome duplication affects biological functions. We found that many genes related to growth, stress response, and cell signaling were more active in polyploids, and identified 2370 important gene regulators grouped in specific regions of the genome. These genes may help explain how polyploid plants adapt and show enhanced traits. Our findings offer useful information for breeding programs that traditionally combine apomictic and sexual seed reproduction, and could lead to new molecular tools to support bahiagrass improvement.

**Abbreviations**
ABA: abscisic acid
ACL: apomixis controlling locus
BUSCO: Benchmarking Universal Single‐Copy Orthologs
DE: differential expression
DET: differentially expressed transcript
FDR: false discovery rate; GC, guanine/citosine;
GO: Gene Ontology
log_2_FC: log_2_ fold change
LOO: leave‐one‐out
KEGG: Kyoto Encyclopedia of Genes and Genomes
NCBI: National Center for Biotechnology Information
PCA: principal component analysis
SRA: Sequence Read Archive
SWIM: SWItch Miner
TF: transcription factor
WGD: whole‐genome duplication

## 1 INTRODUCTION

Polyploidy, the occurrence of multiple sets of chromosomes as a consequence of whole‐genome duplication (WGD), is a recurrent phenomenon in the evolution of major angiosperm lineages, including core eudicots, monocots, orchids, grasses, composites, and legumes (Van de Peer et al., 2017). Polyploidization has played a crucial role in the domestication of numerous crop species by enhancing their genetic diversity and adaptability (Alix et al., 2017; Ha et al., 2009; Heslop‐Harrison et al., 2023; Salman‐Minkov et al., 2016). Increased gene dosage resulting from WGD has been proposed to confer both a genome buffering effect and the possibility for the emergence of allelic novelty, leading to greater resilience to extreme environments (Tossi et al., 2022; Van de Peer et al., 2021). Most plant lineages show evidence of one or a few ancient WGDs that were successfully established (Leebens‐Mack et al., 2019; Wong et al., 2020), and, curiously, their occurrence does not appear to be random, as it often coincides with major stressful periods of global climatic/geologic change and/or mass extinctions (Van de Peer et al., 2021). Polyploidy diversifies gene functions through mechanisms such as subfunctionalization and neofunctionalization, thereby enhancing phenotypic plasticity and adaptive potential in response to environmental challenges (Morris et al., 2024). Stress, polyploidization, and reproductive pathways form a virtuous association. First, stress triggers polyploidy through increased fertilization of non‐reduced gametes (Ramsey & Schemske, 1998). Then, polyploidy confers a selective advantage under stressful or changing environmental conditions (Van de Peer et al., 2017).

In recent years, the subtropical forage grass *Paspalum notatum* has emerged as a suitable model to study the association between polyploidy, reproductive mode, and stress (Ortiz et al., 2013, 2020). In nature, the species is organized as an agamic complex, with diploid (2n = 2x = 20) genotypes reproducing through self‐incompatible sexuality and polyploid ones, mainly tetraploids (2n = 4x = 40), through pseudogamous aposporous apomixis (i.e., asexual reproduction through seeds; Ortiz et al., 2020). Full sexual tetraploids have not been found in nature, but they can be artificially obtained through colchicine‐induced chromosome doubling (Quarin et al., 2001). Moreover, some 2x genotypes can form low proportions of unreduced (2n) female gametophytes (Quarin et al., 2001; Vega et al., 2024), which can occasionally be fertilized by pollen from diploid or tetraploid neighboring plants, resulting in triploid (3x) or 4x progeny, respectively (Daurelio et al., 2004). Therefore, in nature, the genetically divergent 2x populations can occasionally give rise to new hybrid polyploids (triploids and tetraploids), which are often better adapted and capable of founding clonal populations by apomixis, rapidly spreading across the territory, and colonizing demanding habitats (Daurelio et al., 2004).

The strong association between apomixis and polyploidy in *P. notatum* has long triggered interest in characterizing the group of genes whose expression is affected in reproductive organs after polyploidization, which could control key steps of apomixis, such as apospory expressivity and parthenogenesis. In the mid‐2000s, Martelotto et al. (2005) monitored the expression of approximately 10,000 genes in florets of 2x and 4x sexual *P. notatum*, using differential display transcript profiling. The differential expression (DE) of 64 candidate transcripts was validated by reverse‐Northern blot, including several involved in DNA repair, chromatin structure modification, transcription regulation, proteolysis, cell cycle regulation, protein folding, carbohydrate and lipid metabolism, and signal transduction. Moreover, De Oliveira et al. (2020) identified a group of apomixis‐associated candidates by comparing the leaf transcriptomes of apomictic 4x plants with those of sexual 2x and 4x ones, hypothesizing that apomixis‐related genes could be constitutively differentially expressed, not only in ovules.

However, to date, no study has specifically addressed the transcriptomic response of *P. notatum* leaves to polyploidization. To conduct such an analysis, it is essential to compare the leaf transcriptomes of 2x and 4x sexual plants to avoid detecting expression changes derived from the hemizygous genomic region that governs apomixis (i.e., the Apomixis Controlling Locus, or ACL), rather than from ploidy level variation. This approach would allow the unambiguous identification of candidate genes expressed in leaves and specifically associated with ploidy‐related traits, such as stress tolerance or *gigas* effects, which could be leveraged in molecular‐based breeding strategies. Given that *P. notatum* is a major forage grass and has been genetically improved over the past two decades through apomixis‐based breeding programs supported by genomic and transcriptomic markers (Acuña et al., 2019; Marino et al., 2025; Urbani et al., 2017; Zilli et al., 2019), the availability of a comprehensive reference catalog of leaf‐expressed transcripts is crucial. Moreover, the availability of a completely annotated chromosome‐level reference genome of the species (Vega et al., 2024) and robust biolistic transformation techniques (Colono et al., 2019; Mancini et al., 2014, 2018) enables the use of the information contained in this dataset to generate new varieties through genetic engineering. In this study, we used a reference leaf transcriptome previously constructed from available sequencing data (Marino et al., 2025) to map sequences from two diploid sexual (2xsex) and one tetraploid sexual (4xsex) genotypes. As a result, we identified transcripts whose expression is modulated only by the ploidy level. This work provides an inventory of ploidy‐associated genes that can aid in the collaborative data collection from this and other polyploid species, contributing to the characterization of basal transcriptomic responses to increases in the number of genomic complements. Moreover, it uncovers key master regulators involved in complex biological networks, offering valuable targets for breeding programs to enhance yield, stress tolerance, environmental adaptability, and other agronomically important traits.

**Core Ideas**

- Polyploid bahiagrass plants show enhanced adaptation, including stress tolerance and increased vigor.
- Leaf transcriptomes were assembled for sexual diploid and tetraploid bahiagrass plants.
- Transcriptomes reveal how sexual diploid and tetraploid bahiagrass differ at the gene expression level.
- Differentially expressed genes include key breeding targets, such as transcription factors and hormone‐related genes.
- This study provides molecular resources to improve bahiagrass stress tolerance, vigor, and other agronomic traits.

## 2 MATERIALS AND METHODS

### 2.1 Transcriptome assembly and sample clustering

Raw reads derived from transcriptome sequencing of two 2xsex (#22 and #306) and one 4xsex (#216) plants (triplicate biological samples) were downloaded from the National Center for Biotechnology Information (NCBI) database (NCBI BioProject PRJNA476310; De Oliveira et al., 2020). Reads quality‐check was assessed using FastQC (http://www.bioinformatics.babraham.ac.uk/projects/fast<<qc/), and duplicate sequences, ambiguous data, and low‐quality reads were removed using Trim Galore (http://www.bioinformatics.babraham.ac.uk/projects/trim_galore/). Principal component analysis (PCA) and hierarchical clustering were performed to assess sample similarity and quality. High‐quality reads (QC > 30) were reference‐based assembled using the available *P. notatum* R1 genome (NCBI BioProject PRJNA1055536; Vega et al., 2024) and Trinity v2.0.2.

### 2.2 Differential expression assessment

In previous work, we constructed a reference leaf transcriptome of *P. notatum* based on the complete set of raw Sequence Read Archive (SRA) data published by De Oliveira et al. (2020), which were generated from six *P. notatum* accessions with varying ploidy levels and reproductive modes (2xsex: #22 and #306; 4xsex: #216; 4xapo: #115; tetraploid facultative apomictic 4xfac: #34 and #30; DDBJ/EMBL/GenBank accession: DAWXEG000000000, version DAWXEG010000000; Marino et al., 2025). Here, we used this reference leaf transcriptome to quantify expression levels in the 2xsex (#22 and #306), 4xsex (#216), and 4xapo samples (#30, #34, #115), using Kallisto v0.44.0 (https://github.com/pachterlab/kallisto; Bray et al., 2016). Transcript counts were normalized by library size. Transcripts with low counts (fewer than three in all replicates of each library) were filtered out prior to downstream analysis. DE analysis and *p‐*value estimation were conducted using the Bioconductor package DESeq2 (Love et al., 2014). Wald test‐derived *p*‐values were adjusted for multiple testing using the Benjamini–Hochberg method (Benjamini & Hochberg, 1995). Transcripts were considered differentially expressed transcripts (DETs) when the adjusted *p*‐value (*p*_adj_ or FDR) was <0.05 and the absolute log_2_ fold change (|Log_2_FC|) exceeded 2.0. Then, we re‐analyzed the RNA‐seq data using the limma‐voom pipeline (Law et al., 2014) incorporating the duplicateCorrelation function to account for correlations among samples grouped by the ploidy level. The duplicateCorrelation method helped us to reduce false positives that may arise from treating non‐independent genotype samples as independent replicates.

### 2.3 Gene Ontology analysis and KEGG mapping of the DETs

Transcript functional annotation was performed using BLASTn (https://www.ncbi.nlm.nih.gov) against the NCBI NT database. A similar analysis was carried out using the *Arabidopsis thaliana* reference database available at the TAIR website (https://www.arabidopsis.org). Gene Ontology (GO) terms were retrieved using the ClusterProfiler package (Wu et al., 2021; Xu et al., 2024; Yu, 2024, 2012) based on the *Arabidopsis* annotation. Additionally, DETs were subjected to pathway enrichment analysis using the KEGG (Kyoto Encyclopedia of Genes and Genomes) database (https://www.genome.jp/kegg/ko.html). Transcripts were further classified by best single‐directional hits into transcription factor (TF) families using the Plant Transcription Factor Database (https://planttfdb.gao‐lab.org/) and into hormone‐related families using the *Arabidopsis* Hormone Database (https://ngdc.cncb.ac.cn/ahd/browsegene.php). TF family classification was validated using iTAK (Zheng et al., 2016). Gene functional enrichment was also done using MapMan version 3.6.0RC1 (https://mapman.gabipd.org/; Thimm et al., 2004). For functional classification and prediction, DETs were mapped with the Mercator4 webserver (version 7.0; Lohse et al., 2014), and the resulting mapping files were visualized and analyzed in MapMan. Significant DETs (*p*_adj_ ≤ 0.05) and their corresponding log2 fold change values were used as input for alignment and functional categorization within the Mercator framework.

### 2.4 Building gene co‐expression networks

To identify key regulatory genes, an integrated network analysis was performed using the SWItch Miner (SWIM) tool (Paci et al., 2017), which constructs an unweighted correlation network to detect statistically significant master regulators associated with major shifts in the transcriptomic landscape. This study applied SWIM‐based correlation network analysis to predict candidate hub genes within the co‐expression network that may regulate transitions between ploidy states. Correlations were computed across all libraries using Pearson correlations with a James–Stein shrinkage estimator on a genotype‐centered expression matrix (per‐genotype mean subtraction) to treat genotype as a nested blocking factor within ploidy while isolating ploidy‐dependent co‐expression patterns. An adjusted *p*‐value cutoff of 0.05 and a log_2_ fold change (log2FC) threshold of ±1 were used to search for significant nodes. Network robustness was evaluated using leave‐one‐out (LOO) and 80% subsampling analyses, assessing edge preservation (Jaccard), module stability (adjusted Rand index [ARI]), and switch‐gene recovery. To assess whether the switch DETs were randomly distributed across the genome or concentrated in specific regions, we mapped the transcripts onto the diploid reference genome (Vega et al., 2024) and performed a Poisson enrichment test using 1‐Mb genomic bins.

## 3 RESULTS

### 3.1 Sample clustering and transcriptome assembly

**FIGURE 1:**
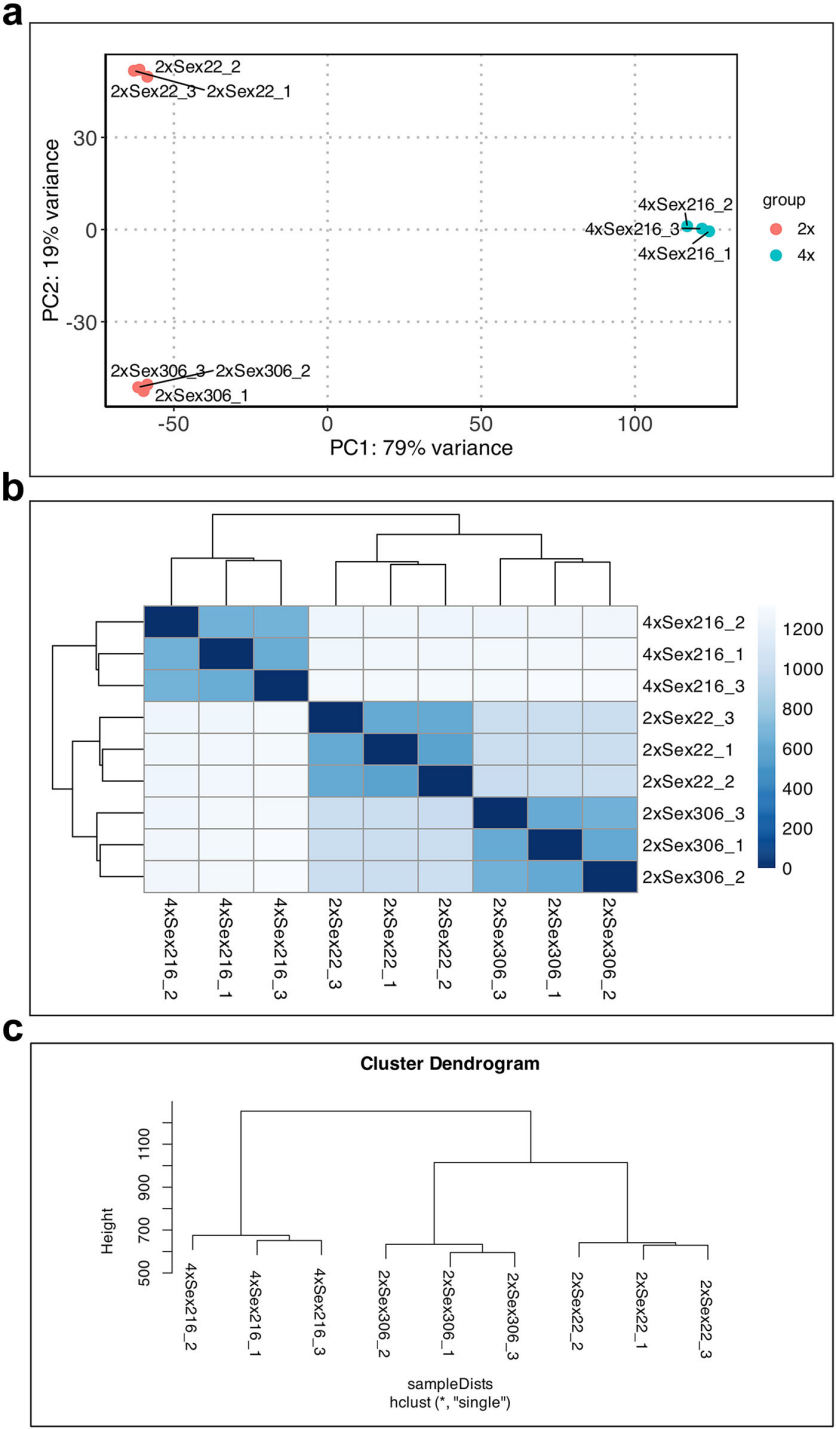
Similarity analysis reveals that *Paspalum notatum* leaf transcriptomes group distinctly according to ploidy level. (a) Principal component analysis (PCA), (b) clustering heatmaps, and (c) dendrograms show the triplicate biological samples used for transcriptome characterization cluster according to ploidy level.

We constructed a leaf diploid transcriptome (2xsex) using two genotypes (#306 and #22) and a tetraploid leaf transcriptome (4xsex) from a single individual (#216). The impute data consisted of raw sequences generated from three biological replicates per genotype, originally produced by De Oliveira et al. (2020) and available in the NCBI Short Read Archive under accession number SRP150615. PCA (Figure 1a) and clustering tests (Figure 1b,c) of the raw data revealed that the replicates were closely grouped and clearly separated according to ploidy level. The resulting transcriptomes were deposited in the NCBI/GenBank repository under accession numbers DAWXED000000000 (2xsex) and DAWXEE000000000 (4xsex). The 2xsex transcriptome assembly contains 76,681 transcripts (contigs) with a GC (guanine/citosine) content of 46.69% and an N50 length of 1545 bp. The 4xsex transcriptome assembly produced 69,996 transcripts (contigs) with a GC content of 46.75% and an N50 length of 1507 bp. The Benchmarking Universal Single‐Copy Orthologs (BUSCO) analysis to evaluate the 2xsex leaf transcriptome completeness revealed 85.26% of complete + partial and 14.74% of the missing genes. Likewise, the BUSCO analysis of the 4xsex leaf transcriptome assemblies showed 79.88% of complete + partial and 20.12% of the missing genes.

### 3.2 Transcripts differentially expressed in leaves of diploid and tetraploid *P. notatum* plants

In previous work (Marino et al., 2025), we constructed a reference leaf transcriptome of *P. notatum* based on the complete set of SRA data published by De Oliveira et al. (2020). This reference transcriptome was established from the data originated from three biological replicates of six genotypes of *P. notatum* with different ploidies and reproductive modes, as follows: 2xsex: genotypes #22 and #306, 4xsex: genotype #216, tetraploid obligate apomict (4xapo): genotype #115, and tetraploid facultative apomict (4xfac): genotypes #34 and #30 (Marino et al., 2025).

**FIGURE 2:**
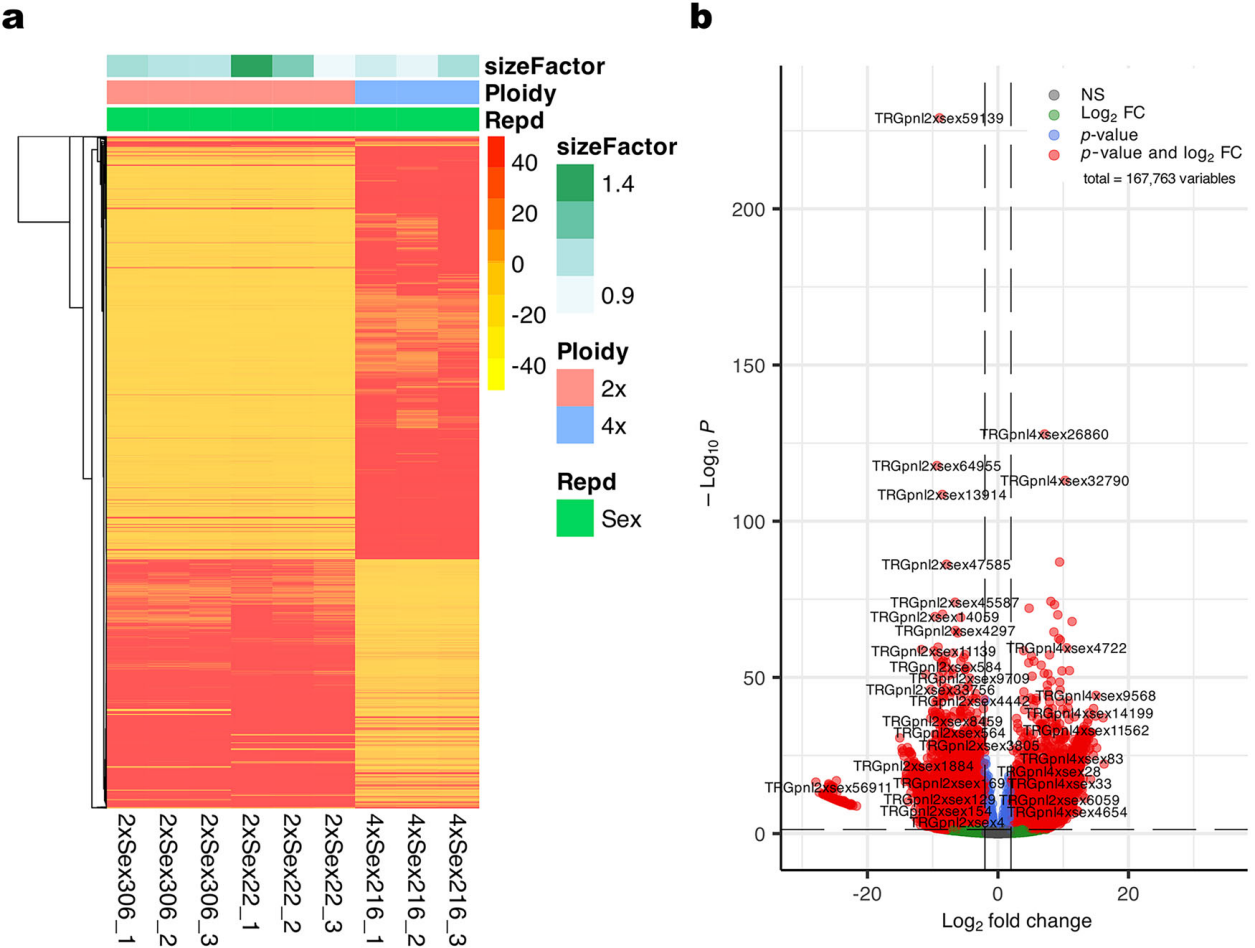
Differentially expressed transcripts (DETs) in leaves of 2x and 4x *Paspalum notatum* genotypes. (a) Heatmaps and (b) volcano plots show a substantial number of transcripts with differential representation between diploid and tetraploid plants.

Initially, we performed a DE analysis between the 2xsex (genotypes #22 and #306) and the 4xsex (genotype #216) samples by mapping the raw sequencing data from the libraries (three biological replicates per genotype) onto the reference transcriptome described above. In this way, we identified 40,844 DETs with highly significant DE (false discovery rate [FDR]: < 0.05, Log_2_FC > |2|) between diploid and tetraploid samples. Of these, 18,785 transcripts were overexpressed in 2xsex (negative log_2_ fold change), while 22,059 transcripts were overexpressed in 4xsex (positive log_2_ fold change). A heatmap illustrating the DETs is presented in Figure 2a, while a volcano plot showing the distribution of log_2_ fold changes is shown in Figure 2b. The complete list of DETs (adjusted *p*‐value < 0.05), including log_2_ fold change values, adjusted *p*‐values, predicted *Arabidopsis* orthologs, and additional annotation data, is provided in Table S1, sheet 1.

### 3.3 Validation of the ploidy effect

To evaluate whether genotype could influence the DESeq2 results, we performed an additional validation analysis including six genotypes: two diploid sexual genotypes (#22 and #306), one tetraploid sexual genotype (#216), and three tetraploid apomictic genotypes (#30, #34, and #115). The DESeq2 design formula was ∼ ploidy + Repd, with ploidy as the contrast. This analysis identified 43,145 DETs. All DETs detected in the initial 2xsex versus 4x sex analysis (40,576) were also present in this expanded validation test (see the Venn diagram and the full list of DETs included in Table S1, sheet 2). These results indicate that the outcome is consistent across genotypes and support the conclusion that the originally observed DETs were associated with ploidy.

Then, we re‐analyzed the RNA‐seq data using the limma‐voom pipeline, incorporating the duplicateCorrelation function to account for correlations among samples grouped by ploidy level in the 2xsex versus 4xsex comparison. This approach helps reduce false positives that may arise when non‐independent genotype samples are treated as independent replicates. The limma‐voom analysis identified 38,576 differentially expressed transcripts, compared with the 40,576 obtained using DESeq2, with an overlap of 28,054 transcripts between both approaches (see Venn diagram and the complete list of overlapped DETs in Table S1, sheet 3). These results indicate that using limma‐voom with duplicateCorrelation provides a more conservative inference of transcripts associated with ploidy level. The 28,054 overlapping DETs were considered “true DETs” and were used for subsequent ontology analyses.

### 3.4 Molecular pathways affected by ploidy increase

DETs were classified according to GO and KEGG ontology terms. Among cellular components, the five most represented categories were the plastid membrane (GO:0042170), outer membrane (GO:0019867), organelle outer membrane (GO:0031968), plastid thylakoid (GO:0031976), and chloroplast thylakoid (GO:0009534), suggesting a notable impact of polyploidy on the photosynthetic apparatus (Figure S1a). Regarding biological processes, the five most affected pathways included protein transport (GO:0015031), regulatory ncRNA processing (GO:0070918), ribonucleoprotein complex biogenesis (GO:0022613), monocarboxylic acid metabolic process (GO: GO:0032787), and leaf development (GO:0048366), indicating that increased DNA content may influence fundamental cellular functions, metabolic activity, and developmental programs in polyploid leaves (Figure S1b). At the molecular function level, DETs were enriched in categories such as protein‐containing complex binding (GO:0044877), ATP hydrolysis activity (GO:0016887), identical protein binding (GO:0042802), phosphoric ester hydrolase activity (GO:0042578), and phosphatase activity (GO:0016791; Figure S1c). The most enriched KEGG pathways included the reductive pentose phosphate cycle (Calvin cycle; M00165), gluconeogenesis oxaloacetate glucose 6‐P (M00003), monolignol biosynthesis phenylalanine tyrosine (M00039), glycolysis core module involving three carbon compounds (M00002), and methionine salvage pathway (M00034), indicating that in polyploid plants central carbohydrate metabolism, lignin biosynthesis, and amino‐acid recycling pathways may be substantially reorganized (Figure S1d).

To gain a comprehensive view of the cell function reprogramming occurring in *P. notatum* following WGD, we used the MapMan approach (version 3.6.0RC1). The main categories affected by WGD included protein homeostasis (1052 DETs), RNA biosynthesis (967 DETs), chromatin organization (404 DETs), lipid metabolism (401 DETs), and photosynthesis (248 DETs; Table S2). Additional evidence of metabolic network remodeling was provided by the enrichment of categories such as RNA homeostasis (343 DETs), amino acid metabolism (216 DETs), carbohydrate metabolism (183 DETs), cellular respiration (179 DETs), and secondary metabolism (138 DETs), which collectively play critical roles in both primary and secondary metabolism (Table S3). Categories related to stress response and signaling were also significantly enriched, including phytohormone action (268 DETs), protein translocation (199 DETs), redox homeostasis (171 DETs), and DNA damage response (90 DETs; Table S4). In particular, within the redox homeostasis category, we observed the deregulation of several enzymes involved in reactive oxygen species scavenging, including superoxide dismutase, catalase, glutathione peroxidase, peroxiredoxin, and ascorbate peroxidase (Table S4; Figure S2). These findings support the well‐established role of polyploidy in enhancing stress tolerance and maintaining cellular homeostasis under both normal and adverse environmental conditions (Das & Roychoudhury, 2014; Sharma et al., 2012).

### 3.5 Transcription factors and hormone‐related transcripts differentially expressed in diploid and polyploid plants

**FIGURE 3:**
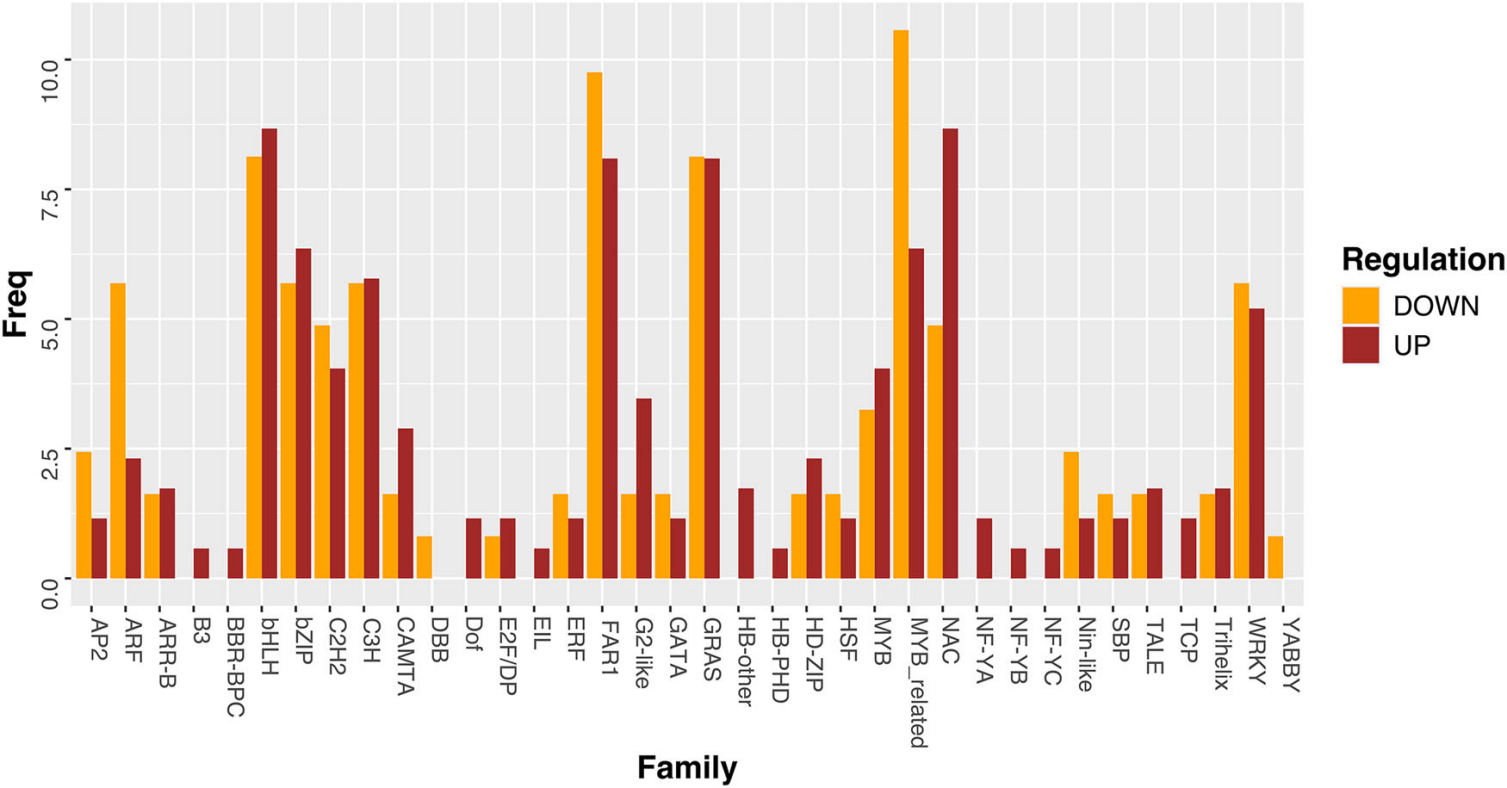
Transcripts encoding transcription factors (TFs) that are differentially expressed in leaves of 2x (control) and 4x *Paspalum notatum* plants. Some TF families were consistently upregulated in 4x plants (B3, BBR‐BPC, Dof, EIL, HB‐other, HB‐PHD, NF‐YA, NF‐YB, NF‐YC, and TCP), while others showed overexpression in 2x plants (DBB and YABBY). The remaining TF families displayed mixed expression patterns, with some members upregulated and others downregulated.

Transcription factors and phytohormones are key physiology regulators and important targets in plant breeding. To investigate the impact of polyploidization on transcriptional regulation in leaves, we analyzed the transcription factor families differentially expressed following WGD. The most prominently affected families included bHLH, bZIP, C2H2, C3H, FAR1, GRAS, MYB‐related, NAC and WRKY, among several others (Figure 3).

**FIGURE 4:**
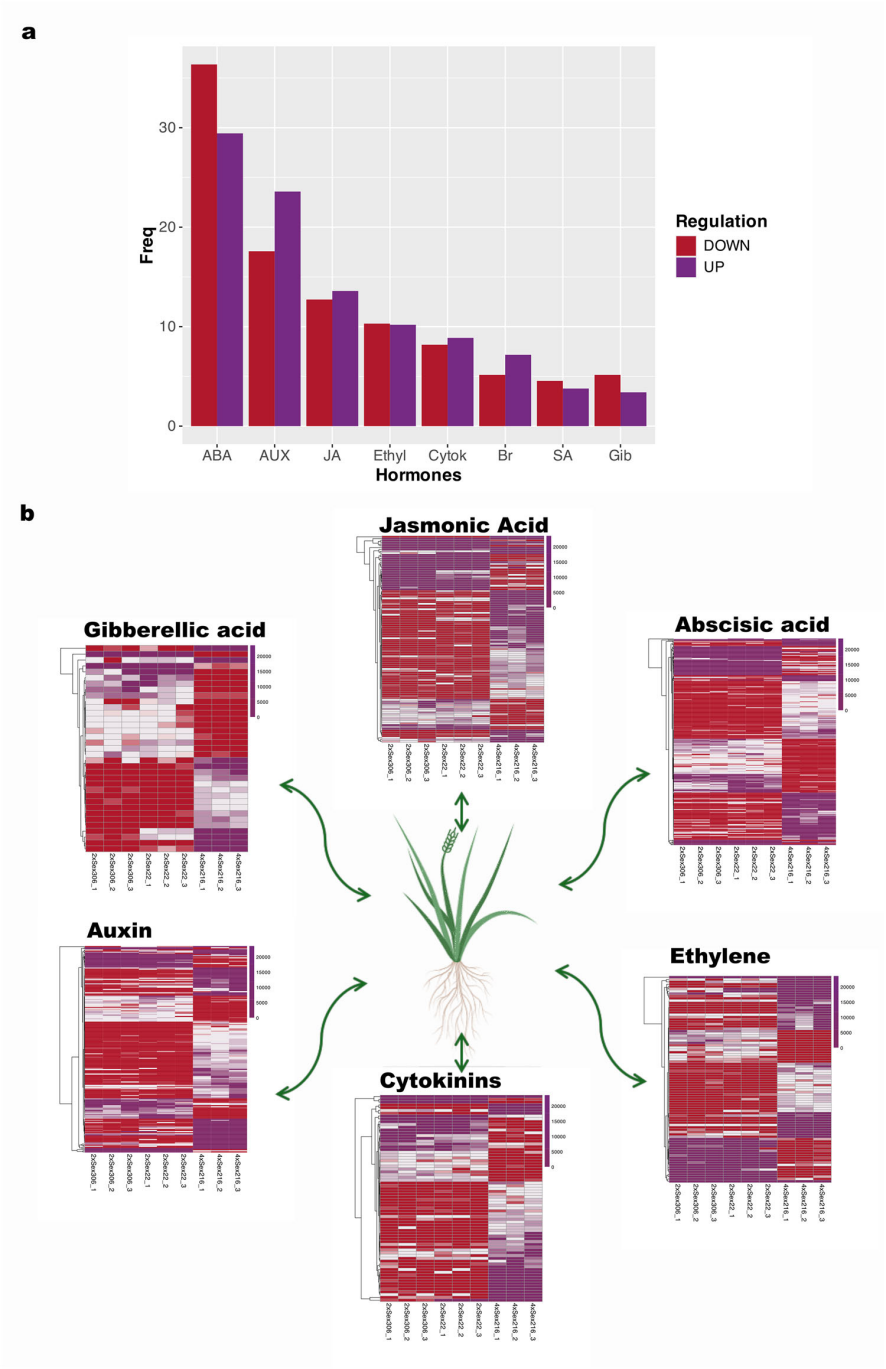
Differential expression of transcripts related to phytohormones in leaves of 2x (control) and 4x *Paspalum notatum* plants. (a) Bar graph showing the main hormone classes affected by ploidy change: abscisic acid (ABA), auxins (AUX), jasmonic acid (JA), ethylene (Ethyl), cytokinins (Cytok), brassinosteroids (Br), salicylic acid (SA), and gibberellins (Gib). (b) Heatmaps showing differential expression across several major hormone‐related categories.

Transcripts involved in hormone synthesis, metabolism, and transport were also identified, with the most prominent categories being abscisic acid, auxin, and jasmonic acid (Figure 4a). Heatmaps clearly illustrate the DE patterns of certain hormone‐related transcript groups, including those associated with gibberellic acid, jasmonic acid, abscisic acid, ethylene, cytokinins, and auxins (Figure 4b). A complete list of all gene transcripts related to transcription factors and hormones is provided in Table S5.

### 3.6 Co‐expression network analysis

**FIGURE 5:**
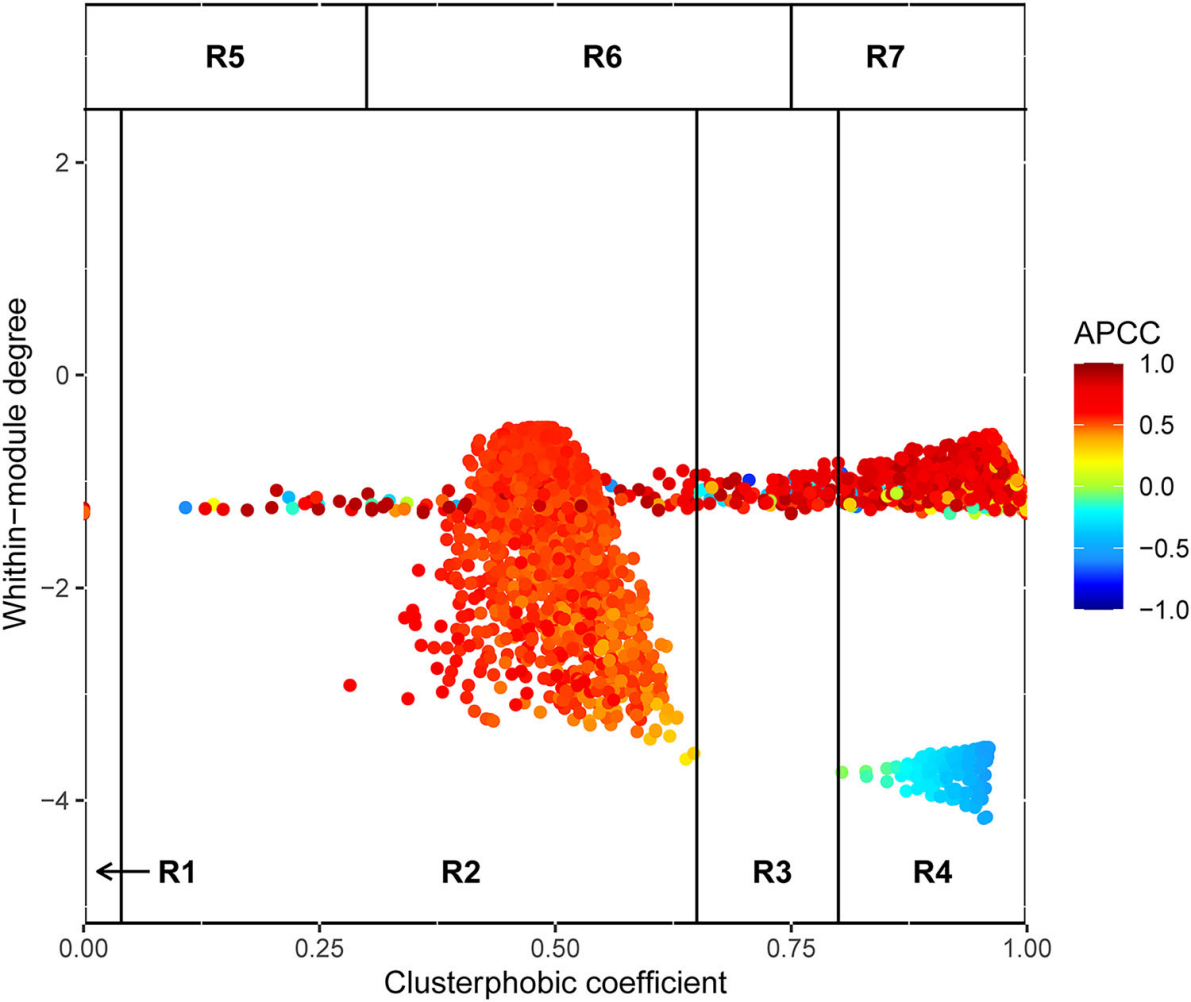
Master regulators affected by genome doubling. SWItch Miner (SWIM) analysis identified hub transcripts differentially expressed between 2x and 4x transcriptomes, with potential regulatory roles in gene expression changes (i.e., switch genes, indicated as light blue dots). APCC, average Pearson correlation coefficient.

SWIM‐based correlation network analysis was conducted to identify master regulators affected by genome duplication (Figure 5). Among the 2835 nodes identified, 532 were classified as switch genes (i.e., hub genes, which are highly connected to other genes in the network and are thought to play a crucial role in regulating the network as a whole; blue dots in Figure 5).

Network robustness was assessed using a shrinkage‐based correlation approach. LOO and 80% subsampling analyses confirmed high network stability (Table 1; Table S6, sheet 1), with median edge Jaccard = 0.80, module ARI = 0.76, and switch‐gene recovery = 0.97 across 109 runs. The consensus switch‐gene set, defined as genes consistently recovered in ≥50% of the runs, included 107 genes, supporting the reproducibility of the switch layer (Table S6, sheet 2).

**TABLE 1:** Summary of network robustness analysis.

Run type	Edge Jaccard	Module ARI	Switch recovery
LOO	0.91 ± 0.03	0.91 ± 0.05	0.98 ± 0.01
Subsample80	0.77 ± 0.12	0.74 ± 0.07	0.94 ± 0.14

*Note*: Edge Jaccard measures edge overlap across runs, module ARI quantifies module assignment consistency, and switch recovery represents the proportion of switch genes consistently identified across runs.

Abbreviations: ARI, adjusted Rand index; LOO, leave‐one‐out.

Many of these switch genes (17) encoded transcription factors, including members of the bHLH, AP2, NAC, RWP‐RK, CCT, GATA, BES1/BZR1, heat stress, APETALA2, and EREBP families, suggesting their involvement in maintaining homeostasis during the transition from 2x to 4x states and in the acquisition of adaptive traits (Table S6, sheet 2). Notably, 14 switch genes were identified as protein kinases, indicating that the network of master regulators induced by WGD may modulate signal transduction processes to control gene expression (Table S6, sheet 2). Additionally, 10 switch genes were specifically associated with the photosynthetic apparatus (e.g., *PsbP*, photosynthetic NDH subunit of subcomplex B1), and 35 switches were identified as stress‐responsive genes (e.g., GST, water stress, and hypersensitive response), which corroborates the findings from the MapMan analysis.

### 3.7 Switch gene hotspots in the *Paspalum notatum* genome

**FIGURE 6:**
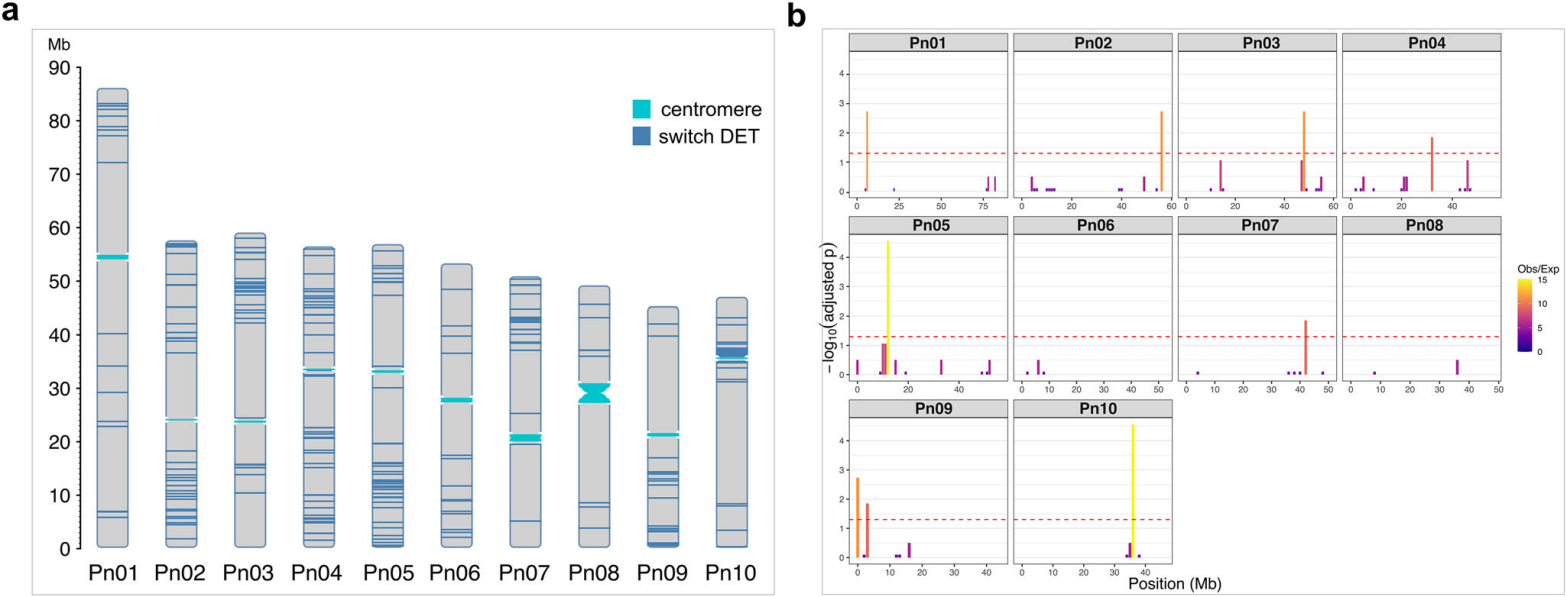
Genomic distribution of differentially expressed master regulators. (a) Switch genes identified by SWItch Miner (SWIM) were mapped across the *Paspalum notatum* genome. (b) A Poisson probability distribution analysis revealed a statistically significant concentration of differentially expressed switch genes in specific genomic regions (hotspots).

To identify potential genomic hotspots containing affected master regulators, we mapped the 532 transcripts classified as switch (i.e., regulatory) network members by SWIM onto the diploid *P. notatum* genome (Figure 6a). A Poisson probability distribution analysis revealed a statistically significant concentration of differentially expressed switch genes in specific genomic regions (hotspots; Figure 6b). The main hotspots were located on chromosome 5 (in a region between 10 and 15 Mb) and on chromosome 10 (in a pericentromeric region between 35 and 40 Mb). Other non‐random hotspots were located in chromosomes 1–4, 7, and 9. These results indicate that changes in ploidy may affect specific genomic regions containing major regulatory genes and therefore have the potential to massively alter expression networks.

## 4 DISCUSSION

*Paspalum notatum*, a major grass component of natural rangelands in South America, is widely used as turf and cultivated pasture in tropical and subtropical regions worldwide (Acuña et al., 2019). The natural occurrence of apomixis (i.e., asexual reproduction through seeds) in this species has enabled the development of novel, rapid, and effective breeding programs, serving as proof of concept for the impact of apomixis on the creation of improved cultivars (Ortiz et al., 2020). These advanced strategies are primarily based on crossing 4x sex mother plants with 4x apo pollen donors, considering that apomictic individuals produce normal, meiotically reduced pollen carrying the apomictic gene, that allow to fix the heterotic combinations in the F_1_ generation (Ortiz et al., 2020). Although 4x sex plants (used as pistillate parents) do not occur naturally, they can be obtained through chromosome doubling of 2x sex plants (Burton et al., 1970; Quarin et al., 2001; Quesenberry et al., 2010). At present, large pools of 4xsex plants are available and ready to capture and recombine the rich allelic diversity locked within asexual highly heterozygous polyploids (Zilli et al., 2019). Furthermore, selecting elite hybrids that inherit full apomixis capacity from the male parent ensures that heterosis can be perpetuated across generations through clonal seed reproduction (Marino et al., 2025; Urbani et al., 2017). These cutting‐edge breeding programs enable the efficient exploitation of genetic resources and allow for the large‐scale multiplication of hybrid genotypes without the need for annual re‐creation, thereby accelerating breeding cycles and significantly reducing seed production costs (Ortiz et al., 2020).

The advanced apomixis‐based breeding schemes can be taken a step further by incorporating molecular tools to assist in selecting superior asexual hybrids. Recently, Marino et al. (2025) provided a successful example of such an approach, applying transcriptome‐guided selection to identify *P. notatum* genotypes with low expression levels of two metabolic genes previously linked to lipid profile modulation: *SUGAR‐DEPENDENT 1* (*SDP1*) and *PEROXISOMAL ABC TRANSPORTER 1* (*PXA1*). This strategy enabled the identification of promising germplasm and the generation/screening of obligate apomictic hybrids with enhanced lipid profiles—namely, increased total lipid content and/or an improved omega‐3 to omega‐6 fatty acid ratio. These improved genotypes could contribute to better milk and meat quality, reducing greenhouse gas emissions associated with grazing (Marino et al., 2025). This pioneering breeding program highlights the importance of having a comprehensive reference leaf transcriptome for the species and a well‐characterized catalog of leaf‐expressed genes, whose expression is associated with the ploidy level, involved in key metabolic and developmental processes of agronomic relevance. Such molecular resources can be instrumental in guiding the development of improved cultivars.

Comparing gene expression profiles between diploid and polyploid genotypes has been proposed as an effective strategy to identify candidate genes potentially involved in regulating adaptive traits (Rosellini et al., 2016; Santoro et al., 2025). Polyploidy has long been recognized as an evolutionary and ecological force driving the adaptation of plant species to stressful or demanding environments (Van de Peer et al., 2021). In the context of climate change, identifying key master regulators involved in genome‐level responses to polyploidization is particularly relevant, as it could enable the molecular selection of genotypes with enhanced adaptation to diverse environmental conditions. Furthermore, the occurrence of *gigas* effects, accelerated metabolism, increased growth vigor, and elevated production of secondary metabolites in polyploids (Van de Peer et al., 2021; Yali, 2022) offer a unique opportunity to identify genes controlling traits critical for forage production. Recent experiments conducted in rice have shown that polyploidy alters the expression of genes involved in sugar and metal transport (Ghouri et al., 2023). RNA‐seq analyses identified several differentially expressed genes between polyploid and diploid rice, particularly those encoding metal and sucrose transporters, as well as ploidy‐specific pathways associated with plant growth and development (Ghouri et al., 2023). Moreover, the transcriptomic, biochemical, and physiological responses of rice to abiotic stress—particularly salt and cadmium stress—were reported to be considerably enhanced in tetraploid plants compared with diploids, revealing an increased representation of specific transcripts related to stress tolerance (Sun et al., 2025).

In this study, we provide a comprehensive catalog of genes expressed in the leaves of 2x sexual and 4x sexual genotypes and identify those that are up‐ or downregulated following genome duplication. Initially, a large number (40,844) of DETs showing highly significant differential expression (FDR < 0.05; |log_2_FC| > 2) were identified by comparing two 2x sexual and one 4x sexual genotype. This set of DETs was fully validated by a second analysis using the data from three additional 4x plants, in this case apomictic ones. Subsequently, a smaller refined set of DETs (28,054) was obtained using the limma‐voom pipeline, which helped reduce false positives that may arise when treating non‐independent genotype samples as independent replicates. The transcripts belonging to this reduced set of DETs identified after the limma‐voom analysis were considered “true DETs” and were included in subsequent analyses.

Ontology analysis revealed an enrichment of categories typically associated with polyploid phenotypes, including photosynthesis, cell cycle, and stress responses. The availability of this list, alongside the reference transcriptome (Marino et al., 2025), reference genome (Vega et al., 2024), and an optimized transformation platform for the species (Colono et al., 2019; Mancini et al., 2014, 2018), enables the design of targeted strategies for molecular‐assisted breeding and/or genetic engineering. Based on the premise that polyploidy often enhances yield, stress tolerance, environmental adaptability, and other agronomically important traits—such as asexual reproduction by seeds (i.e., apomixis)—the lists of annotated DETs detected in different organs of 2x and 4x plants, as reported here, may highlight specific gene candidates to be prioritized for functional analyses and, subsequently, for molecular breeding. The identification of DETs associated with known biological functions and pathways could foster further investigations into the effects of their overexpression or repression through genetic engineering in diploid contexts. This strategy may contribute to improving the agronomic performance of elite diploid genotypes. For instance, assuming that polyploids exhibit higher yields and gigas effects, some of the cell‐cycle–related transcripts differentially expressed between 2x and 4x plants could be targeted in genetic engineering experiments to reproduce, in diploid backgrounds, phenotypes typically observed in tetraploids. Such an approach could facilitate the pyramiding of new desirable traits onto already improved diploid genetic backgrounds.

Given the key roles of TFs and hormone‐related transcripts in regulating stress responses and developmental pathways, we focused on identifying these categories among the DETs. As expected, one of the most prominent classes of affected transcription factors was the NAC family—a group of plant‐specific TFs known to play crucial roles in diverse aspects of plant development and stress adaptation (Xiong et al., 2025). NACs orchestrate complex plant growth and resilience strategies and have recently been proposed as promising targets for engineering stress‐resistant crops and enhancing agricultural productivity (Xiong et al., 2025). With respect to phytohormones, in addition to the five classical ones—auxins, cytokinins, gibberellins, abscisic acid (ABA), and ethylene—others such as brassinosteroids, jasmonic acid, and salicylic acid also play central roles in plant growth and stress responses. All these classes were represented within the DET list obtained in our analysis. Moreover, a model describing the integrated action of multiple phytohormones in plants under drought conditions, based on ABA responses in leaf tissue, was proposed by Zhao et al. (2021). Drawing on both this model and our list of candidate genes would help design targeted breeding strategies for *P. notatum* to develop cultivars with enhanced stress tolerance.

Interestingly, when we focused on the subset of master regulatory genes within the pool of differentially expressed transcripts, we observed that these regulators were not randomly distributed across the *P. notatum* genome. Instead, many of them appeared to cluster in specific genomic regions. These hotspots may represent critical areas that either harbor key orchestrators of the transcriptomic response or are particularly sensitive to the effects of WGD. The accumulation of master regulators in such regions suggests the existence of genomic hubs that coordinate complex biological pathways during the transition from 2x to 4x ploidy level, including those related to stress adaptation and developmental modulation. This observation opens new avenues for exploring genome organization in relation to regulatory control and provides a foundation for precision breeding efforts. Altogether, our findings contribute to a deeper understanding of the transcriptomic consequences of genome duplication in *P. notatum* and highlight a set of candidate genes and regulatory hotspots that can be prioritized in future molecular breeding or genome editing initiatives aimed at improving agronomically desirable traits such as stress tolerance, productivity, and resilience in this and other agronomically important species.

## AUTHOR CONTRIBUTIONS

**Maricel Podio**: Formal analysis; methodology; writing—review and editing. **Danilo Fabrizio Santoro**: Formal analysis; methodology; writing—review and editing. **Carolina Marta Colono**: Conceptualization; writing—review and editing. **Juan Pablo A. Ortiz**: Conceptualization; writing—review and editing. **Emidio Albertini**: Conceptualization; funding acquisition; writing—review and editing. **Silvina Claudia Pessino**: Conceptualization; funding acquisition; writing—original draft; writing—review and editing.

## CONFLICT OF INTEREST STATEMENT

All authors declare no conflicts of interest.

## Supporting information




**Supplemental Figure S1**: Dot plots showing differentially expressed transcripts (DETs) classified according to GO and KEGG ontology terms.

**Supplemental Figure S2**: Transcripts classified within the Redox Homeostasis category by MapMan.

**Supplemental Table S1**: List of differentially expressed transcripts (DETs) in leaves of 2x and 4x *Paspalum notatum* plants (adjusted p‐value < 0.05), including the associated log_2_ fold‐change values, adjusted p‐values, and predicted *Arabidopsis orthologs*. The table includes the DET sets derived from the 2x sex vs. 4x sex comparison, the 2x sex vs. all 4x validation analysis, and the limma‐voom analysis.

**Supplemental Table S2**: List of differentially expressed transcripts (DETs) involved in major biological categories affected by whole genome duplication (WGD): protein homeostasis, RNA biosynthesis, chromatin organization, lipid metabolism, and photosynthesis.

**Supplemental Table S3**: List of differentially expressed transcripts (DETs) related to primary and secondary metabolism, including RNA homeostasis, amino acid metabolism, carbohydrate metabolism, cellular respiration, and secondary metabolism.

**Supplemental Table S4**: List of differentially expressed transcripts (DETs) involved in stress response and signaling processes: phytohormone action, protein translocation, redox homeostasis, and DNA damage response.

**Supplemental Table S5**: List of differentially expressed transcripts (DETs) related to transcription factors and hormone signaling.

**Supplemental Table S6**: SWIM‐based network robustness assessment and putative switch genes identified by SWIM among the differentially expressed transcripts in 2x and 4x *Paspalum notatum* plants.

## Data Availability

The raw sequence data used to construct the 2x sex and 4x sex transcriptomes and to perform all DE analyses are available in the NCBI SRA repository, under accession number SRP150615 (de Oliveira et al., 2020). The 2xsex and 4xsex transcriptome assemblies are available in the NCBI/GenBank repository, accession numbers DAWXED000000000 and DAWXEE000000000, respectively. The reference transcriptome assembly used for DE analysis is available in the NCBI/GenBank repository, accession number DAWXEG000000000 (the version used in this article was DAWXEG000000000.1; Marino et al., 2025). This published article and its Supporting Information files include the rest of the data generated or analyzed during this study.
