# Supplementary figures and images for: Leaf transcriptome differences between diploid and tetraploid bahiagrass
Source: Plant Genome. 2026 Feb 28;19(1):e70212. doi: 10.1002/tpg2.70212 (PMC12949672; doi:10.1002/tpg2.70212)

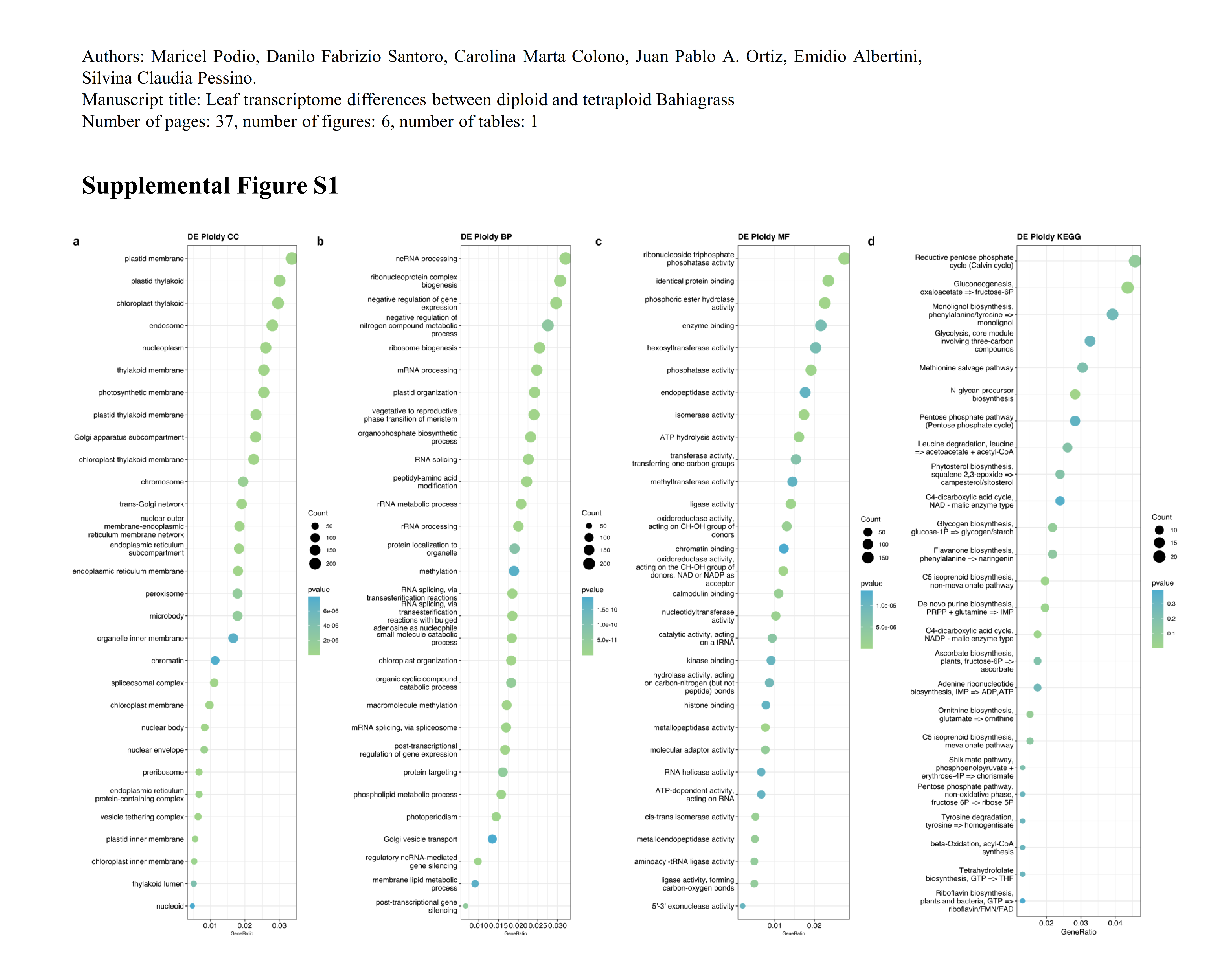

Supplement: Supplementary file 1 — Supplemental Figure S1: Dot plots showing differentially expressed transcripts (DETs) classified according to GO and KEGG ontology terms. [file TPG2-19-e70212-s002.tif]

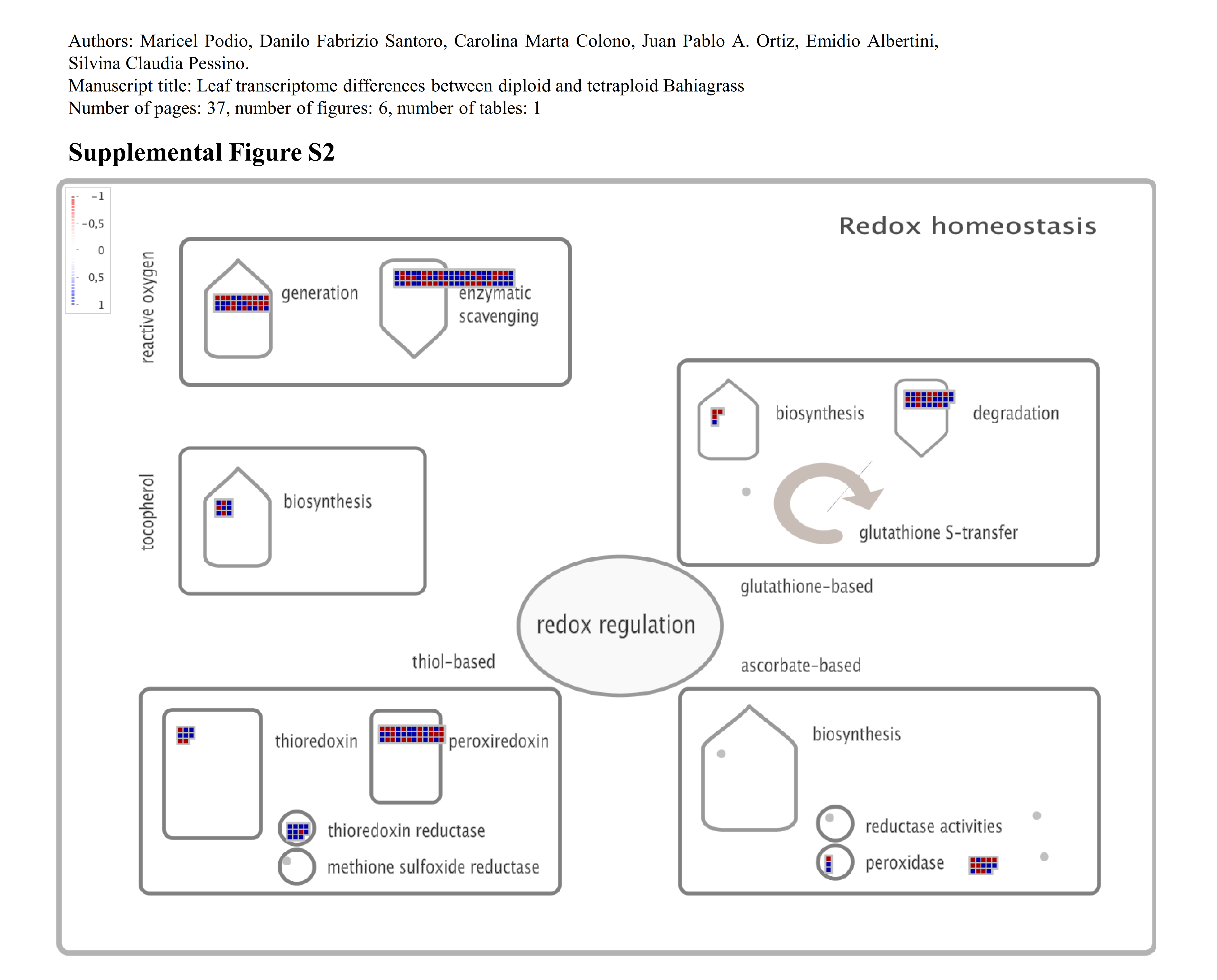

Supplement: Supplementary file 2 — Supplemental Figure S2: Transcripts classified within the Redox Homeostasis category by MapMan. [file TPG2-19-e70212-s003.tif]
